# Erythrodermic pityriasis rubra pilaris following SARS-CoV-2 vaccination treated with bimekizumab

**DOI:** 10.1016/j.jdcr.2023.09.024

**Published:** 2023-10-04

**Authors:** Mysa Saad, Reetesh Bose

**Affiliations:** a Faculty of Medicine, University of Ottawa, Ottawa, Ontario, Canada; b Division of Dermatology, the Ottawa Hospital, Ottawa, Ontario, Canada

## Introduction

SARS-CoV-2 vaccinations have been linked to several cutaneous adverse effects, including psoriasiform dermatoses, such as pityriasis rubra pilaris (PRP).^1^, ^2^, ^3^ PRP is a rare inflammatory papulosquamous disorder of unclear etiology. The interleukin (IL) 23/Th17 axis is thought to play a role in the pathophysiology of PRP.^4^ There have been 2 reported cases of PRP following vaccination with the mRNA-1273 (Moderna) COVID-19 vaccine.^3^^,^^5^ Most vaccine-induced PRP cases have been effectively treated with retinoids or corticosteroids. Ixekizumab and ustekinumab have also been reported in the literature as successful therapies for SARS-CoV-2 vaccine-induced PRP.^1^^,^^3^^,^^6^ We report a case of new-onset severe erythrodermic PRP after the Moderna SARS-CoV-2 vaccination, treated with selective IL-17A and IL-17F inhibition.

## Case report

**Fig 1:**
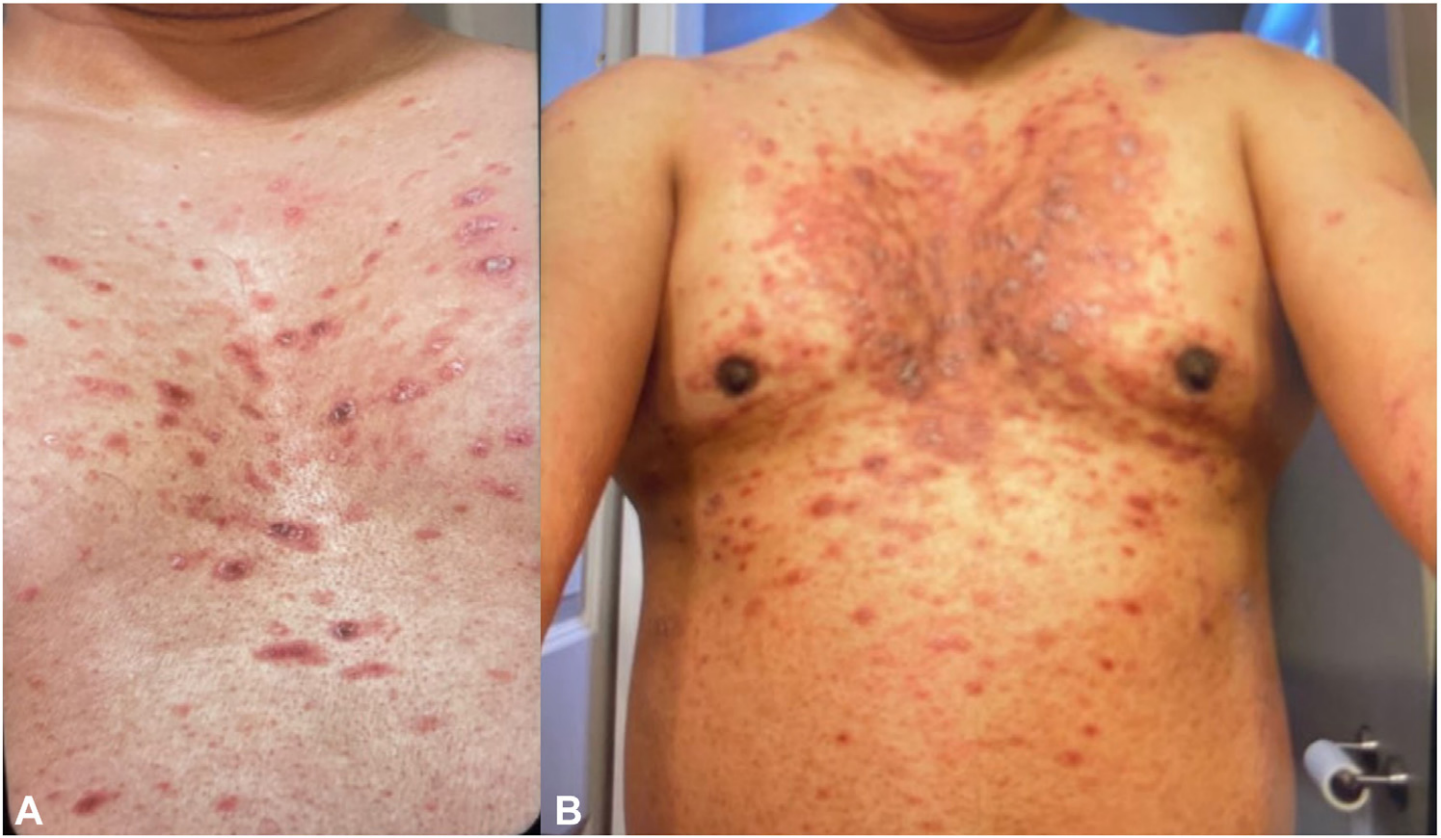
Patient-provided photographs of the central trunk demonstrating (**A**) localized red oval macules and papules with central heme crusting and overlying silver scale on February 15, 2022 (1 month following Moderna vaccination) and (**B**) red macules and papules coalescing into large plaques on February 28, 2022.

A 42-year-old man with a remote history of dry scalp and hand dermatitis presented with a painful, pruritic, and widespread rash. The rash first appeared approximately 1 month after receiving the Moderna vaccine in January 2022. The rash was initially localized to the trunk with red oval macules and papules coalescing into large plaques with overlying, nutmeg grater-like scale and central hemorrhagic crust (Fig 1, *A* and *B*). No other inciting event was identified prior to the development of this rash. He had negative HIV and COVID-19 tests. He had received the Pfizer-N-BioTech SARS-CoV-2 vaccine in June 2021, followed by the Moderna vaccine in July 2021, with no associated adverse effects. After his third SARS-CoV-2 vaccine (January 2022), which was his second dose of Moderna vaccine, the rash rapidly progressed to involve >90% of his body surface area, with focal islands of sparing on his trunk and extremities and desquamation of the digits.

**Fig 2:**
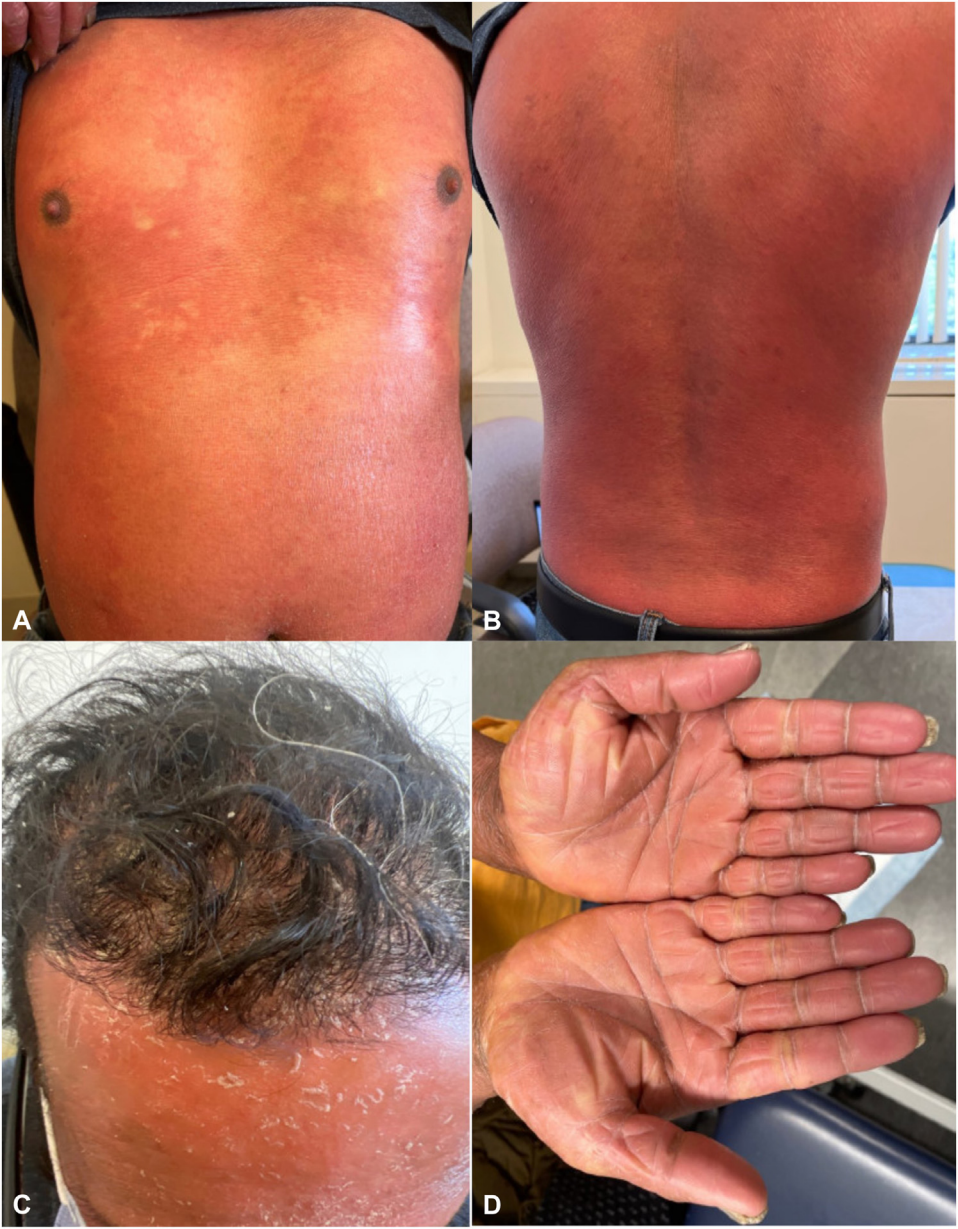
Diffuse erythroderma affecting (**A**) the entire abdomen and chest, (**B**) the entire back, (**C**) erythroderma of the face and scalp with desquamative scale. **D,** Bilateral palms demonstrating orange-waxy keratoderma.

Dermatology was consulted and when assessed, he was completely erythrodermic with diffuse desquamative scale and marked orange-waxy palmoplantar keratoderma (Fig 2, *A-D*). He later developed subungual hyperkeratosis, subungual hemorrhage, and onychomadesis involving all fingernails and toenails. Initial biopsies revealed a psoriasiform and spongiotic dermatitis with acanthosis, spongiosis, compact hyperkeratosis and parakeratosis, and superficial perivascular lymphocytic inflammation. Repeat biopsies of the forearm and abdomen confirmed a psoriasiform dermatosis.

Additionally, he endorsed systemic symptoms, including exertional dyspnea with peripheral edema, and unintentional over 40-pound weight loss over a 6-month period. Echocardiogram and computed tomography scan of the chest were unremarkable. Sezary syndrome was ruled out based on negative flow cytometry and lack of T-cell receptor gene rearrangement. On bloodwork, anti-Sjogren's syndrome-related antigen A antibody was positive; however, anti-double stranded DNA and anti-Smith antibodies were negative. Multiple biopsies did not show any signs of lupus erythematosus or connective tissue disease.

**Fig 3:**
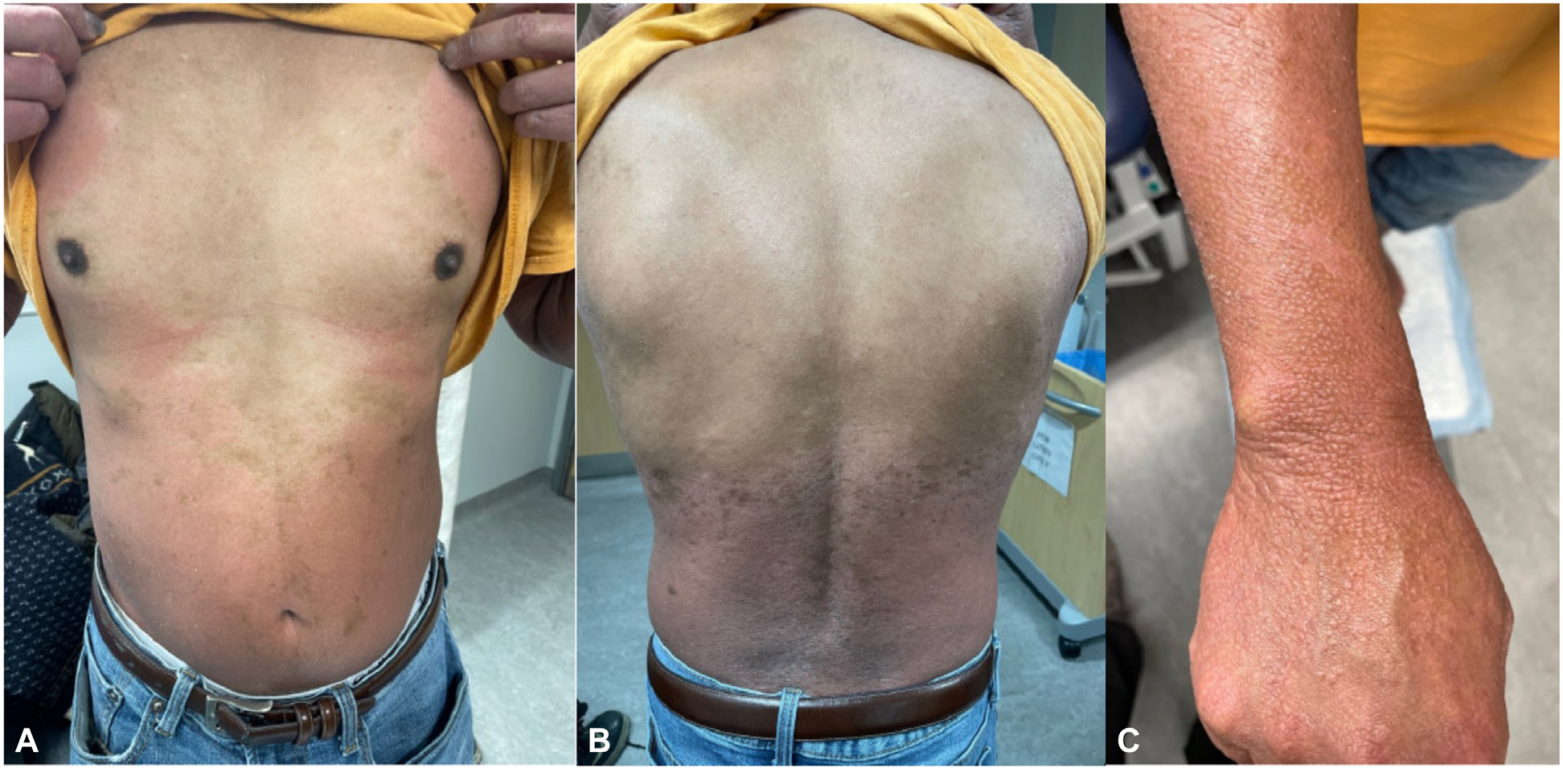
Red/orange erythematous patches with follicular prominence and islands of sparing on the (**A**) trunk, abdomen, and (**B**) back in January 2023. **C,** Nutmeg grater-like scale and follicular papules on the dorsal aspect of the forearm and wrist. Four months after starting bimekizumab.

**Fig 4:**
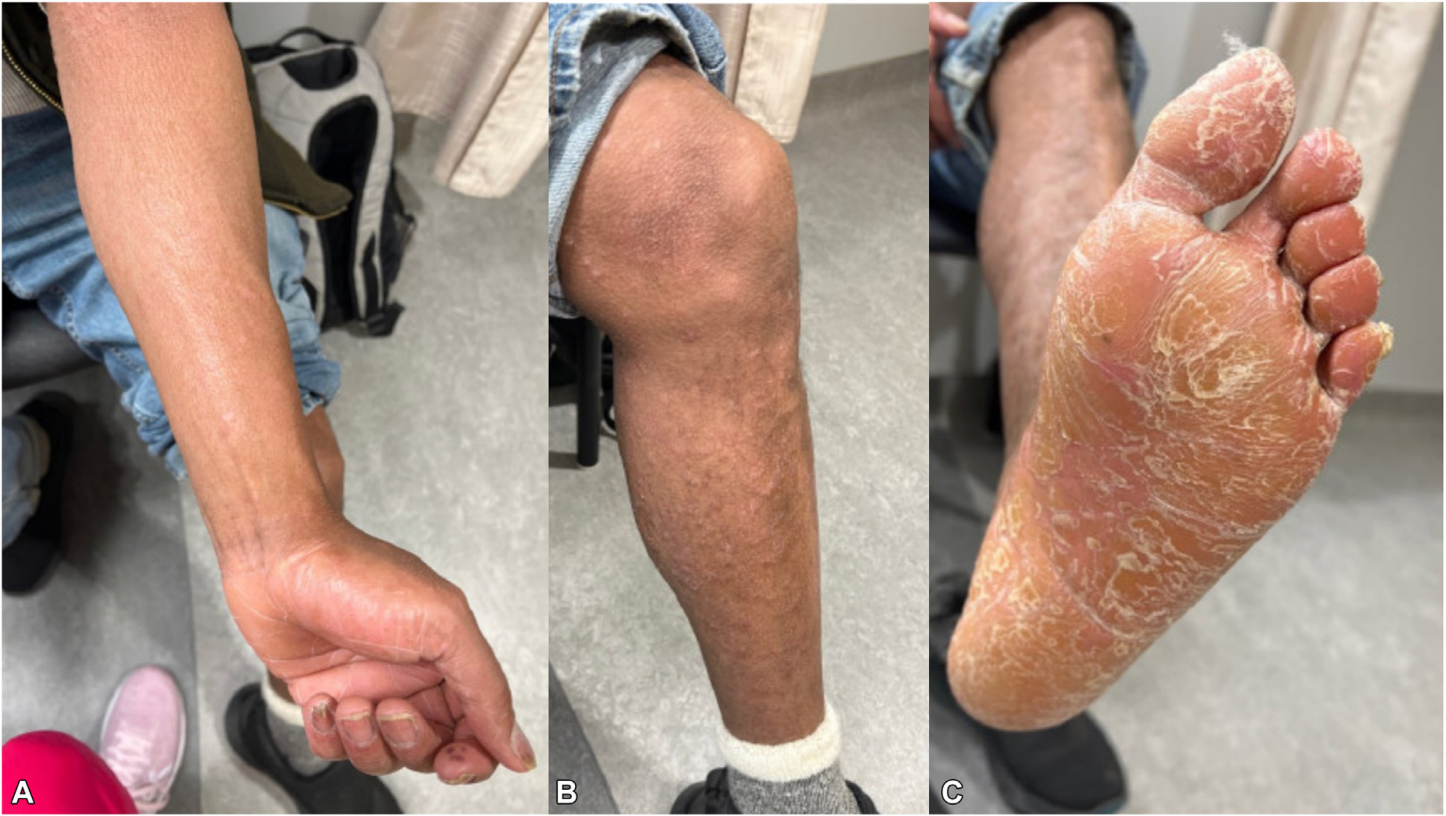
Resolved erythroderma in May 2023, 8 months after starting bimekizumab; **A,** flat-topped follicular papules without overlying scale on the forearm, orange-waxy palm with significantly improved hyperkeratosis, ongoing nail dystrophy with resumption of normal nail growth; **B,** follicular papules without overlying scale on the lower portion of leg; **C,** hyperkeratosis of the plantar aspect of the foot with lamellar scaling.

He had been treated with 2 courses of oral prednisone (35 mg daily and 40 mg daily for 5 days plus tapering) and topical betamethasone 0.1% lotion with no significant improvement. A 6-week trial of cyclosporine (150 mg twice daily for 1 month, followed by 200 mg twice daily for 2 weeks) exacerbated his rash and was discontinued. This finding would be atypical for psoriasis. In September 2022, 6 months after rash onset, he was started on bimekizumab 320 mg subcutaneously every 4 weeks, then transitioned to injections every 8 weeks as maintenance dosing. He was noted to have significant improvement in the erythroderma on his trunk and back, with persistent nutmeg grater-like scale and follicular papules on the dorsal aspect of the forearms and wrists in January 2023 (Fig 3, *A-C*). By May 2023 (8 months after starting bimekizumab), his erythroderma had resolved, with only follicular papules remaining on the forearms, hands, and lower portion of the legs. His palmoplantar keratoderma and hyperkeratosis also significantly improved, with normal nail growth (Fig 4, *A-C*).

He was thought to either have a very severe form of psoriasis, or PRP. Given the nutmeg grater-like scale, follicular papules, orange-waxy palmoplantar keratoderma, islands of sparing, and lack of response to prednisone or cyclosporine, PRP was favored.

## Discussion

Since the global launch of COVID-19 vaccination campaigns, numerous cutaneous adverse effects have been reported, including PRP and erythrodermic psoriasis. Our case contributes to this expanding body of literature. This was a severe case of erythrodermic PRP with systematic manifestations, refractory to several usual treatment lines. Given the latency of exposures, the most likely trigger for this severe reaction was the Moderna vaccine. This is supported by other case reports, which describe similar latency periods of up to 4 weeks between COVID-19 vaccination and onset of PRP.^7^^,^^8^ The pathophysiology behind new-onset PRP following COVID-19 vaccination is unclear. However, the patient’s dramatic clinical presentation may point to an underlying genetic susceptibility that was environmentally precipitated.

Although PRP can spontaneously resolve, this was unlikely as the patient only began to improve after starting treatment with bimekizumab. Fortunately, the patient experienced near total improvement within 8 months of treatment with bimekizumab, an IL-17A and IL-17F antagonist approved for the management of moderate-to-severe psoriasis and psoriatic arthritis. Evidence regarding the use of IL-17A antagonists for PRP is limited to 2 single-arm trials and case reports.^9^^,^^10^ To our knowledge, there have been no other reports of the successful use of bimekizumab for the treatment of PRP or vaccine-induced PRP. The patient’s favorable response to bimekizumab reinforces the implication of the IL-23/Th17 axis in the pathogenesis of PRP and suggests a promising role for these agents in the management of this condition.

In conclusion, it is important for clinicians to be aware of the rare but significant link between SARS-CoV-2 vaccination and PRP as well as the potential therapeutic options, including IL-17A inhibitors. Further research is required to elucidate the possible mechanisms underlying this association and to determine the most effective therapeutic agent for this condition.

## Conflicts of interest

None.

## References

[1] Zhao P., Rusu C.A., Schenck O.L. (2023). Pityriasis rubra pilaris following COVID-19 vaccination successfully treated with ixekizumab. JAAD Case Rep.

[2] Sahni M.K., Roy K., Asati D.P., Khurana U. (2021). An old entity, a new trigger: post COVID-19 vaccine pityriasis rubra pilaris. Int J Risk Saf Med.

[3] Ajebo E.M., Howard J.D., Anand D., Davis L.S. (2022). Pityriasis rubra pilaris potentially triggered by messenger RNA–1273 COVID vaccine. JAAD Case Rep.

[4] Feldmeyer L., Mylonas A., Demaria O. (2017). Interleukin 23-helper T cell 17 axis as a treatment target for pityriasis rubra pilaris. JAMA Dermatol.

[5] Sechi A., Pierobon E., Pezzolo E. (2022). Abrupt onset of Sweet syndrome, pityriasis rubra pilaris, pityriasis lichenoides et varioliformis acuta and erythema multiforme: unravelling a possible common trigger, the COVID-19 vaccine. Clin Exp Dermatol.

[6] Gambichler T., Scheel C.H., Arafat Y., Kautz O., Boms S. (2022). Erythrodermic pityriasis rubra pilaris after SARS-CoV-2 vaccination with concomitant COVID-19 infection. J Eur Acad Dermatol Venereol.

[7] Bramhoff A.C., Wesselmann U., Bender S.T., Berghoff A.V., Hofmann S.C., Balakirski G. (2022). [Pityriasis rubra pilaris after COVID-19 vaccination: causal relationship or coincidence?]. Dermatologie (Heidelb).

[8] Fernández L.T., Pérez-Garza D.M., delaO-Escamilla A. (2022). Pityriasis rubra pilaris in association with inactivated SARS-CoV-2 vaccine (CoronaVac). Dermatol Ther.

[9] Haynes D., Strunck J.L., Topham C.A. (2020). Evaluation of ixekizumab treatment for patients with pityriasis rubra pilaris: a single-arm trial. JAMA Dermatol.

[10] Boudreaux B.W., Pincelli T.P., Bhullar P.K. (2022). Secukinumab for the treatment of adult-onset pityriasis rubra pilaris: a single-arm clinical trial with transcriptomic analysis. Br J Dermatol.

